# Chimeric Human Papillomavirus-16 Virus-like Particles Presenting P18I10 and T20 Peptides from HIV-1 Envelope Induce HPV16 and HIV-1-Specific Humoral and T Cell-Mediated Immunity in BALB/c Mice

**DOI:** 10.3390/vaccines11010015

**Published:** 2022-12-21

**Authors:** Chun-Wei Chen, Narcís Saubi, Athina Kilpeläinen, Joan Joseph-Munné

**Affiliations:** 1 Department of Biomedical Sciences, University of Barcelona, 08036 Barcelona, Spain; 2 Vall d’Hebron Research Institute, 08035 Barcelona, Spain; 3 Respiratory Viruses Unit, Virology Section, Microbiology Department, Vall d’Hebron Hospital Universitari, Vall d’Hebron Institut de Recerca (VHIR), Vall d’Hebron Barcelona Hospital Campus, Passeig Vall d’Hebron 119-129, 08035 Barcelona, Spain; 4 Department of Microbiology, Hospital Universitari Vall d’Hebron, 08035 Barcelona, Spain

**Keywords:** HIV-1, HPV16, vaccine, virus-like particles, P18I10, T20 enfuvirtide, BCG.HIVA, humoral immunity, T cell-mediated immunity

## Abstract

In this study, the HIV-1 P18I10 CTL peptide derived from the V3 loop of HIV-1 gp120 and the T20 anti-fusion peptide of HIV-1 gp41 were inserted into the HPV16 L1 capsid protein to construct chimeric HPV:HIV (L1:P18I10 and L1:T20) VLPs by using the mammalian cell expression system. The HPV:HIV VLPs were purified by chromatography. We demonstrated that the insertion of P18I10 or T20 peptides into the DE loop of HPV16 L1 capsid proteins did not affect in vitro stability, self-assembly and morphology of chimeric HPV:HIV VLPs. Importantly, it did not interfere either with the HIV-1 antibody reactivity targeting sequential and conformational P18I10 and T20 peptides presented on chimeric HPV:HIV VLPs or with the induction of HPV16 L1-specific antibodies in vivo. We observed that chimeric L1:P18I10/L1:T20 VLPs vaccines could induce HPV16- but weak HIV-1-specific antibody responses and elicited HPV16- and HIV-1-specific T-cell responses in BALB/c mice. Moreover, could be a potential booster to increase HIV-specific cellular responses in the heterologous immunization after priming with rBCG.HIVA vaccine. This research work would contribute a step towards the development of the novel chimeric HPV:HIV VLP-based vaccine platform for controlling HPV16 and HIV-1 infection, which is urgently needed in developing and industrialized countries.

## 1. Introduction

Human immunodeficiency virus-1 (HIV-1), which causes acquired immunodeficiency syndrome (AIDS), was discovered in the early 1980s, and since then it has become a global epidemic [1]. Although highly active anti-retroviral treatment (HAART), together with pre-exposure prophylaxis (PrEP) can have a real impact on the control of HIV-1 infection, vaccination is still a fundamental approach for public benefit and to put an end to global HIV-1 epidemic [2]. In spite of over three decades of thorough HIV-1 research and numerous vaccine clinical trials, a licensed HIV-1 vaccine until now is still unachievable. The RV144 trial conducted in Thailand was the first case to reveal a modest efficacy of 31.2% against acquisition of HIV-1 infection [3]. The majority of other HIV-1 vaccine candidates that underwent clinical trials were mainly based on DNA, recombinant viral vectors or subunit protein models [4,5]. Ideally, an efficacious HIV-1 vaccine is capable of inducing innate immune responses, neutralizing antibodies to prevent viral infection [6] as well as cytotoxic T lymphocytes (CTL) responses to eliminate infected cells [7]. However, eliciting each response may require different vaccine strategies, warranting separate but parallel development efforts. The selection of immunogens and delivery vectors will have significant impacts on function and specificity of HIV-1 vaccines [8,9]. The repeated failures using the standard approaches for the HIV-1 vaccine development led to a recognition of the importance of delivery vector selection, prime-boost regimes and immunogen specificity in both humoral and cellular responses. More than 100 types of human papilloma virus (HPV) are already known and the HPV genotypes 16 and 18 are considered to be responsible for approximately 70% of cervical cancers worldwide [10]. HPV L1 virus-like particles (VLPs), classified as a type of subunit vaccines, could predominantly induce comparable L1-specific humoral responses to wild-type virion and also T cell-mediated responses [11,12,13]. Currently, three HPV preventive vaccines have been licensed on the market and all of them are based on VLPs of HPV L1 capsid protein. Two of them, Gardasil (Merck, Rahway, NJ, USA) and Gardasil-9 (Merck) are produced by the yeast (Saccharomyces cerevisiae) expression system while the other, Cervarix (GSK, Brentford, UK), is produced by the baculovirus expression vector/insect cell (BEVS/IC) system [14]. Until now, optimum conditions of production HPV16 L1 proteins in the mammalian expression system have not been well-established. Thus, the development of a combined vaccine that would protect against HPV and HIV infections is a logical effort in the fight against these two major global pathogens.

In our previous review paper publication regarding design concepts of virus-like particle (VLP)-based HIV-1 vaccines, we mentioned that non-enveloped VLPs, such as papillomavirus VLPs, could play a functional role as delivery vectors to present HIV-1 CTL or neutralizing antibody epitopes [15,16]. This hypothesis has been confirmed in several chimeric bovine papillomavirus (BPV) L1 VLP presenting P18I10 CTL epitope from V3 loop of gp120 HIV-1 envelope protein (Env) and 2F5 epitope or MPER region of gp41 HIV-1 Env [17,18,19,20,21,22]. The structural feature of human papillomavirus type-16 (HPV16) L1 capsid proteins is similar to that of BPV and could self-assemble into single-layer L1 VLPs [23]. Five of the HPV16 L1 proteins form a pentamer and 72 of the pentamers self-assemble into an HPV16 VLP [24]. However, there is still no clear evidence that chimeric HPV16:HIV capsid proteins could be stable in vitro and self-assemble into morphologically integral VLPs. On the other hand, HPV16 L1 VLPs have been demonstrated to be highly immunogenic and are capable of inducing antigen-specific T and B-cell immune responses [11,12,13]. It still remains to be seen whether the presentation of HIV-1 epitopes through HPV:HIV VLPs could be immunogenic.

In this study, we aimed at developing a chimeric VLP-based HPV:HIV vaccine by using human 293F cells, a well-established mammalian cell expression system. The HPV 16 L1 protein acted as the structural vaccine scaffold, and the P18I10 and T20 peptides were selected as HIV-1 immunogens and inserted into the DE loop of HPV16 L1 protein. The immunodominant P18I10 CTL epitope comprising 10 amino acids (residues 311–320: RGPGRAFVTI) is derived from the third variable domain (V3) of the HIV-1 envelope glycoprotein gp120. The P18I10 peptide has been identified as a H-2Dd-restricted MHC class-I molecule to induce cytotoxic T lymphocytes (CTL) responses [25,26]. The T20 peptide, known as Enfuvirtide and designed as an antiretroviral multimeric fusion peptide, consists of a 36 amino acid sequence (YTSLIHSLIEESQNQQEKNEQ ELLELDKWASLWNWF) mimicking the C-terminal heptad helix sequence close to the membrane′s proximal external region (MPER) of the HIV-1 envelope glycoprotein 41 (gp41) [27]. These two HIV-1 T (P18I10) and B (T20) cell-based epitopes were selected as a starting point and proof of concept experiment for the chimeric VLP-based HPV:HIV vaccine development platform.

Over the past decade, many different prime-boost formats of VLP-based HIV-1 vaccine have been tested [15]. Although the majority of previous HIV-1 VLP [28] or chimeric BPV:HIV VLP [17,18,19,20,21,22] vaccine strategies were focused on inducing immune responses by using the homologous prime-boost regimen, two former studies suggested that heterologous immunization consisting of recombinant *Mycobacterium bovis* Bacillus Calmette-Guérin (rBCG) expressing HIV-1 Gag prime and HIV-1 Gag VLP boost may contribute to enhance T-cell immunity [29,30]. In our research group, we have demonstrated that priming with rBCG expressing HIVA immunogen and boosting with recombinant viral vector MVA.HIVA was safe and elicited HIV-1-specific T-cell immune responses in BALB/c mice [31,32,33]. The HIVA immunogen, designed by Dr. Tomas Hanke, is composed of the full-length HIV-1 Gag protein combined with multiple CTL epitopes including P18I10 epitopes at the C-terminus [34]. Therefore, we aimed to evaluate whether rBCG.HIVA could boost the T-cell immune responses induced by HIV:HPV (L1:P18I10) VLPs in BALB/c mice.

In this study, the chimeric HPV:HIV (L1:P18I10 and L1:T20) immunogens were designed and produced by using 293F expression system. The chimeric L1:P18I10 and L1:T20 protein expression was confirmed by immunostaining. The HPV:HIV VLPs were subsequently purified by a 3-step chromatographic method, including cation (CEC), size exclusion (SEC) and heparin affinity (H-AC) chromatography. Then, the in vitro stability, in vitro self-assembly and morphology of purified HPV:HIV VLPs were confirmed by non-reducing SDS-PAGE, molecular mass assay and transmission electron microscopy (TEM), respectively. The sequential and conformational P18I10 and T20 peptides presented on chimeric HPV:HIV VLPs were further characterized by anti-HIV-1 gp120 V3 and 2F5 monoclonal antibodies in vitro by using Western blot and indirect ELISA assay. Finally, the immunogenicity of HPV:HIV VLPs was assessed in BALB/c mice model. We demonstrated that chimeric L1:P18I10 and L1:T20 VLP-based vaccines could induce HPV16- and HIV-1-specific antibody responses and chimeric L1:P18I10 VLPs could induce HPV16- and HIV-1-specific T-cell responses in BALB/c mice. Because the development and manufacturing of an immunogenic HPV:HIV vaccine is still unachievable, this study provided a baseline strategy that may be worth supporting the global efforts to develop novel chimeric VLP-based vaccines for controlling HPV and HIV-1 infections.

## 2. Materials and Methods

### 2.1. Construction of the BCG.HIVA^2auxo.int^ Vaccine Strain

Recombinant BCG expressing HIVA immunogen was previously constructed using the *E.coli*-mycobacteriol integrative shuttle vector p2auxo.int. The construction of *E. coli*/mycobacterial vector expressing HIVA antigen was previously described [31,32,33]. BCG.HIVA^2auxo.int^ was diluted in PBS-Tween20 to 2 × 10^7^ cfu/mL, sonicated to disrupt bacterial clumps and inoculated into the rear food pad or BALB/c mice (50 µL, 10^6^ cfu/mouse).

### 2.2. Bacterial Cultures and Transformation

Cells of the glycine auxotrophic strain of *E. coli*, M151GlyA (Invitrogen, Waltham, MA, USA), provided by Dr. Pau Ferrer, were cultured in minimal M9-derivative medium (M9-D: Na_2_HPO_4_, 6.78 g/L; KH_2_PO_4_, 3 g/L; NaCl, 0.5 g/L; NH_4_Cl, 1 g/L, glucose, 10 g/L; MgSO_4_, 2 mmol/L; CaCl_2_, 0.1 mmol/L; thiamine, 0.1 g/L; FeCl_3_, 0.025 g/L; AlCl_3_·6H_2_O, 0.13 mg/L; ZnSO_4_·7H_2_O, 2.6 mg/L; CoCl_2_·6H_2_O, 0.47 mg/L; CuSO_4_·H2O, 4.6 mg/L; H_3_BO_3_, 0.03 mg/L; MnCl_2_·4H_2_O, 4.2 mg/L; NiCl_2_·6H_2_O, 0.02 mg/L; Na_2_MoO_4_·2H_2_O, 0.06 mg/L), supplemented with glycine (70 μg/mL). The *E. coli* M151Gly cells were transformed with the p2auxo.HIVA plasmids by electroporation. For this, the *E. coli* cultures were grown to an optical density of 0.9 at 600 nm, transformed using a Bio-Rad gene pulser electroporator at 2.5 kV, 25 μF, and 200 Ω. The transformed cells were subsequently cultured on M9-D agar plates (components as previously described, with 1.5% bactoagar added) without glycine supplementation for selection or with glycine supplementation as a control. The lysine auxotrophic BCG strain, BCGΔ*lys*, kindly provided by W.R. Jacobs Jr., B.R. Bloom, and T. Hsu was transformed with p2auxo.HIVA^int^ plasmid DNA by electroporation. The mycobacteria were cultured in Middlebrook 7H9 broth medium or on Middlebrook agar 7H10 medium supplemented with albumin-dextrose-catalase (ADC; Difco) containing 0.05% Tween 80. L-lysine monohydrochloride (Sigma, Kawasaki, Japan) was dissolved in distilled water and used as a supplement at a final concentration of 40 μg/mL. For transformation, BCG was cultured to an optical density of 1.5 at 600 nm, transformed using a Bio-Rad gene pulser electroporator at 2.5 kV, 25 μF, and 1000 Ω. The transformants were then cultured on ADC-supplemented Middlebrook agar 7H10 medium containing 0.05% Tween 80 without lysine supplementation.

### 2.3. Cell Lines and Cell Culture

The 293F cells (gibco), derived from human embryonic kidney (HEK) 293 cells, were cultured in FreeStyle 293 expression medium (gibco) supplemented with 5 mL/L of penicillin-streptomycin (gibco) and incubated in a 37 °C incubator containing a humidified atmosphere of 5% CO_2_ on an orbital shaker platform rotating at 125 rpm.

### 2.4. Production of L1:P18I10 and L1:T20 Proteins Using the 293F Expression System

The pCDNA3.1 construct contained L1:P18I10 or L1:T20 DNA coding sequences corresponding to chimeric L1:P18I10 and L1:T20 proteins, respectively. The HIV-1 P18I10 CTL peptide (RGPGRAFVTI) or T20 peptide (YTSLIHSLIEESQNQQEKNEQE LLELDKWASLWNWF) were inserted into the DE loop of HPV16 L1 capsid protein. The HPV16 L1 DE loop sequence encoding 130–136 amino acids was replaced with either P18I10I10 or T20 peptide. The L1:P18I10 or L1:T20 DNA coding sequences were modified with Kozak sequence, optimized with human codon, flanked by the restriction enzyme sites of HindIII and XbaI and cloned into pcDNA3.1(+) vector by using GeneArt gene synthesis services (Thermo Fisher, Waltham, MA, USA). The recombinant plasmid DNA (pDNA) was transformed into *E. coli* DH5α competent cells (Invitrogen) for amplification and extracted by using plasmid Maxi kits (QIAGEN, Hilden, Germany). The 293F cells were cultured with 30mL FreeStyle 293 expression medium in a 125mL Erlenmeyer flask (Corning, New York, NY, USA) to a density of 1.0 × 10^6^/mL and transiently transfected with L1:P18I10 or L1:T20 pDNAs using the branched polyethylenimine with a MW of 25 kDa (PEI-25K) (Polysciences) at an optimized ratio of DNA to PEI 1:3 (*w*/*w*) and DNA to culture medium 1:1 (*w*/*v*), according to manufacturer’s instructions [35]. The 293F cells were harvested at 96 h post-transfection. The 293F cells can reach a confluent density of 3.6 × 10^6^ cells/mL with approximately 50% viability.

### 2.5. Immunofluorescence Staining

The cells were permeabilized on the glass slide with 100% cold acetone. Subsequently, the fixed cells were probed with anti-HPV16 L1 antibody CAMVIR-1 (Abcam, Cambridge, UK) and captured with anti-mouse IgG-FITC (Sigma). Immune-stained cell monolayers were thoroughly washed with PBS and covered with mounting medium with DAPI (Abcam). The immunofluorescence images were inspected under an inverted microscope at 40× magnification. Transfection efficiency was determined by the ratio of FITC (green)-positive cells to DAPI (blue)-stained cells.

### 2.6. Purification of HPV:HIV (L1:P18I10 and L1:T20) VLPs

A total of 10^8^ transfected 293F cells in a 125 mL Erlenmeyer flask (30 mL culture medium/flask) were collected by centrifugation at 1500 rpm for 5 min and washed twice with PBS. Cell pellets were resuspended in lysis buffer formulated with 1% Triton X-100, protease inhibitor (1:100) (Millipore) and Benzonase (25 U/mL) (Millipore). Cell lysates were clarified with 0.45 µm PVDF syringe filter (Millipore). The HPV:HIV (L1:P18I10 and L1:T20) VLP samples were serially purified using cation exchange (Capto SP ImpRes, GE, Boston, MA, USA), size exclusion (Capto Core 700, GE) and affinity (HiTrap Heparin HP, GE) chromatography. The chromatographic protocols were described in our previous studies [36,37] and following the manufacturer’s protocol [38]. The L1 protein signal in each purification step was characterized by Western blot analysis and probed with anti-HPV16 L1 antibody CAMVIR-1 [39].

### 2.7. Non-Reducing SDS-PAGE

The HPV16 L1, L1:P18I10 and L1:T20 VLPs were mixed with 2× Laemmli sample buffer (BIO-RAD) in the absence or presence of 5% (*v*/*v*) 2-mercaptoethanol (2-ME) and reacted at room temperature (RT) for 24 h. Samples were separated by 8–16% TGX stain-free protein gels (BIO-RAD). Then, the gels were transferred to PVDF membranes. The membranes were probed with the anti-HPV16 L1 CAMVIR-1 mAb at a dilution of 1:4000. After that, the membranes were incubated with anti-mouse IgG Peroxidase Conjugate (Sigma-Aldrich, St. Louis, MO, USA) at a dilution of 1:4000. The signal was developed and visualized by chemoluminiscence using Western Blot ECL substrate kit (Bio-Rad, Hercules, CA, USA). The blot images were acquired by using Odyssey Fc imaging system.

### 2.8. Molecular Mass Analysis

The HPV16 L1, L1:P18I10 and L1:T20 VLPs without 2-ME treatment were filtered out through 1000 kDa molecular weight cutoff (MWCO) ultrafiltration devices (SARTORIUS). The HPV16 L1, L1:P18I10 and L1:T20 VLPs with 2-ME treatment were passed through 100 kDa MWCO ultrafiltration devices (Amicon). The retentates were reconstituted to the original volume and collected from the filter device sample reservoir, while the filtrates were collected at the bottom of the centrifuge tube. The L1 signal was measured by using dot blot probed with anti-HPV16 L1 mAb and detected by anti-mouse IgG-peroxidase conjugate (Sigma-Aldrich). Images were acquired using Odyssey Fc Imaging System at a chemiluminescence channel.

### 2.9. Negative Staining and Transmission Electron Microscopy

After charging the carbon-coated copper grids (Sigma-Aldrich) under ultraviolet light for 5 min, commercial HPV16 L1 (Abcam), purified L1:P18I10 and L1:T20 VLPs equilibrated with 20 mM Tris-HCl (pH 7.4, 137 mM NaCl) were absorbed on grids for 1 min and rinsed three times by miliQ water. The HPV:HIV VLPs were negative-stained with 2% uranyl acetate at pH 4.5 (Sigma-Aldrich) for 1 min. Excess staining agents were removed by Whatman qualitative filter paper (Sigma-Aldrich). Grids were placed in a dehumidifier chamber at least 2 h before observation. Images were acquired using a transmission electron microscope (Tecnai Spirit 120 kV) at magnification SA135K (100 nm) and SA59000 (200 nm), respectively.

### 2.10. Sodium Dodecyl Sulfate–Polyacrylamide Gel Electrophoresis and Western Blotting Analysis

Equal amounts (500 ng) of HPV16 L1 protein (Abcam), purified L1:P18I10 and L1:T20 VLPs were mixed with 2× Laemmli sample buffer containing 5% 2-ME and boiled at 95 °C for 5 min. Samples were separated by 8–16% TGX Stain-free protein gels and then transferred to a PVDF membrane (Millipore, Burlington, MA, USA) using a Semi-Dry transfer device (Bio-Rad). The membrane was blocked with 5% skim milk in TBST. Then, the membranes were probed with the anti-HPV16 L1 CAMVIR-1 mAb at a dilution of 1:4000, anti-HIV-1 gp120 V3 loop mAb (NIBSC, EVA3012) at a dilution of 1:40 and HIV1 gp41 (2F5) mAb (NIBSC, ARP3063) at a dilution of 1:4000, respectively. After that, the membranes were incubated with anti-mouse IgG Peroxidase Conjugate (Sigma-Aldrich) at a dilution of 1:4000. The Western ECL substrate kit (BIO-RAD) was used for signal development. The blot images were acquired by using Odyssey Fc imaging system at a chemiluminescence channel.

### 2.11. Immunization of Mice, Collection of Sera and Isolation of Splenocytes

This is a preliminary proof-of-concept study to demonstrate immunogenicity of HPV:HIV VLPs (L1:P18I10 and L1:T20 VLPs). The dose, administration route and prime-boost interval of our HPV:HIV VLPs referred to previous studies that immunized mice with bovine papillomavirus (BPV):HIV VLPs for inducing antibody responses [20,21]. Purified HPV:HIV VLPs were emulsified with an equal volume of (225 μg per each 0.5 mL dose) aluminum hydroxyphosphate sulfate (Thermo Fisher), to ensure a similar formulation to the licensed Gardasil-9 HPV vaccine [40]. All mouse groups had equal gender distribution (male *n* = 4 and female *n* = 4 per group). In groups A and B, BALB/c mice were immunized intramuscularly (i.m.) with 10 μg of L1:P18I10 or L1:T20 VLPs, respectively, by following a homologous prime-boost regime. In the group C, mice were inoculated with 10^6^ cfu of BCG.HIVA^2auxo.int^ intradermally (i.d., at the food pad) and boosted with 10 μg of L1:P18I10 VLPs intramuscularly. In the group D, positive control mice were inoculated with Gardasil-9 prime followed by Gardasil-9 boost intramuscularly with 10 μg of HPV16 L1 VLPs. In the group E, negative control mice were immunized twice with PBS buffer. The prime-boost interval was 2 weeks. Mice were sacrificed on day 28. Blood samples were collected from the heart of mice. Sera were recovered by centrifugation and stored at −20 °C for ELISA assay. Murine spleens were removed and pressed individually through a cell strainer (Falcon) with a 5 mL syringe rubber plunger. Following the removal of red blood cells with ACK lysing buffer (Lonza), splenocytes were washed and resuspended in lymphocyte medium R10 (RPMI 1640 supplemented with 10% fetal calf serum (FCS), penicillin-streptomycin, 20 mM HEPES and 15 mM 2-ME) at a concentration of 2 × 10^7^ cells/mL.

### 2.12. Enzyme-Linked Immunosorbent Assay

To test the HPV16 L1- and HIV-1-specific antibodies binding to chimeric HPV:HIV VLP constructs in vitro, 50 µL of equal concentration (200 ng/mL) of recombinant HPV16 L1 protein (Abcam, ab119880), purified L1:P18I10 and L1:T20 VLPs in 50mM carbonate-bicarbonate buffer (pH = 9.6) (Sigma) were 2-fold serially diluted and coated onto the Maxisorb plates (Nunc). The plates were incubated at 4 °C overnight. Plates were blocked with the blocking buffer (5% skim milk in TBST) at 37 °C for at least 2 h. After wash twice with TBST, the VLP-coated plates were incubated with anti-HPV16 L1 CAMVIR-1 mAb at a dilution of 1:8000, 2F5 mAb (NIBSC, ARP3063) at a dilution of 1:8000 and anti-HIV-1 gp120 V3 loop mAb (NIBSC, EVA3012) at a dilution of 1:40 in blocking buffer, respectively, at 37 °C for 2 h. After washing three times with TBST, the plates were incubated with recombinant protein G peroxidase conjugate (Thermo Scientific, Waltham, MA, USA) at a dilution of 1:4000 in blocking buffer at 37 °C for 1 h. TMB was used to develop the enzyme-linked immunosorbent assay (ELISA) signal and stopped with 50 µL of 2M H_2_SO_4_. The optical density (OD) of each well was measured and recorded at a wavelength of 450 nm by using EL × 800 absorbance microplate reader.

To measure the VLP-induced antibodies in BALB/c mice, the microtiter plates were coated with 50 µL of 2 µg/mL recombinant HPV16 L1 protein (Abcam, ab119880), HIV-1 P18I10 peptide (NIBSC, ARP734), T20 peptide (NIBSC, ARP984), respectively, with 50 mM carbonate-bicarbonate buffer (pH = 9.6). The plates were incubated at 4 °C overnight. Plates were blocked with the blocking buffer (5% skim milk in TBST) at 37 °C for at least 2 h. At the same time, sera collected from the group A-E immunized mice were diluted with 5% skim milk in TBST at a ratio of 1:50. After wash twice with TBST, the plates were incubated with the diluted sera at 37 °C for 2 h. After washing three times with TBST, the plates were added with recombinant protein G HRP conjugate at a dilution of 1:4000 in blocking buffer and incubated at 37 °C for 1 h. TMB was used to develop the ELISA signal and stopped with 50 µL of 2M H_2_SO_4_. The OD of each well was measured at a wavelength of 450 nm by using EL × 800 absorbance microplate reader (Biotek).

### 2.13. IFN-γ ELISpot Assay

The enzyme-linked immune absorbent spot (ELISpot) assay was performed using the commercial murine IFN-γ ELISpot kit (Mabtech, Nacka Strand, Sweden), according to the manufacturer’s instructions. The ELISpot plates (MSISP4510, 96-well plates with polyvinylidene difluoride membranes, Millipore, Middlesex County, MA, USA) were 70% EtOH treated and coated with purified anti-mouse interferon-γ (IFN-γ) capture monoclonal antibody diluted in phosphate-buffered saline (PBS) to a final concentration of 5 µg/mL at 4 °C overnight. Then, 2.5 × 10^5^ fresh splenocytes were added to each well. Subsequently, the cells from groups A and B were stimulated with 2 μg/mL of HPV16 L1 VLPs and HIV-1 P18I10 peptides, respectively. All the samples and controls were plated in duplicate wells. ELISpot assays were incubated for 16 h at 37 °C, 5% CO_2_. The plates were subsequently washed 5 × with PBS, incubated for 2 h with a biotinylated anti-IFN-γ monoclonal antibody (mAb) diluted in PBS 2% Fetal Calf Serum (FCS) to a final concentration of 2 µg/mL, washed 5 times in PBS, and incubated with the streptavidin-alkaline phosphatase conjugate in PBS 2% FCS. Then, plates were washed 5 times with PBS before incubating with 100 µL of 5-bromo-4-chloro-3-indolyl phosphate (BCIP)/nitro blue tetrazolium (NBT) substrate solution (Sigma-Aldrich, St. Louis, MO, USA). After 5–10 min, the plates were washed with tap water, dried, and the resulting spots counted using an ELISPOT reader (AID, Autoimmun Diagnostika GmbH, Strasberg, Germany). For each animal, the mean of background responses was subtracted individually from all the wells to enable a comparison of the IFN-γ spot forming cells (SFC)/10^6^ between groups. To define positive responses, a threshold was defined as at least five spots per well, and responses exceeding the mean number of spots in negative control wells plus three standard deviations of the negative control wells.

### 2.14. Statistical Analysis

All statistical analysis was performed using Prism 6 GraphPad software (CA, USA). We used data from experiments carried out over 5 different ELISA plate coating concentration (0, 50, 100, 150, 200 ng/mL) of recombinant HPV16 L1 protein (Abcam, ab119880) and HPV:HIV VLPs. We collected data from 2 groups (HPV16 L1 and HPV:HIV VLPs) and collected three replicates for each coating concentration. The graph in Prism showed the data as a scatterplot showing coating concentration (ng/mL) on the X-axis and OD450 on the Y-axis. Since we hypothesized that our in vitro ELISA data is related in a linear pattern, we performed the linear regression analysis and compared the slopes of the two lines to confirm that data set-1 and data set-2 (antibody reactivity between HPV16 L1 and HPV:HIV VLPs) are different in their actions. We selected 95% confidence intervals alongside the best-fit line. Prism was automatically overlaid a linear regression line on both our data sets and it has plotted a dotted line representing 95% confidence intervals. During the linear regression analysis, the software calculated only the mean Y value of our data set that indicated show the goodness of fit. In addition, Prism gave us a commentary, so we can conclude that the differences between two slopes are extremely significant.

We had 5 sets (ELISA) and 4 sets (ELISPOT) of data collected from mouse immunization experiments. These data sets had an equal number (male *n* = 4 and female *n* = 4) in each group. We used Gardasil-9-immunized mice as positive control group and PBS-immunized mice as negative control group. The data of our designed experiments were not matching or pairing. We undertook one-way analysis of variance (ANOVA) to see whether these means were different. We first checked our data fit Gaussian normal distribution. We found that some groups of data in ELISA assay did not past the Gaussian normal distribution test. Therefore, we carried out a non-parametric statistical analysis on these data. By contrast, all groups of data in the ELISPOT assay past the Gaussian normal distribution test. Thus, we preformed a parametric statistical analysis on these data. The significance threshold and confidence level were set to 0.05 (equivalent to 95% confidence interval). *p* value showing in general the probability that there are differences between groups. Since our main concern for this experiment is what are the differences between our groups, the multiple comparisons in Prism gave us the outcome of comparison in all groups with every other group.

### 2.15. Ethics Statements

Six to eight-week-old BALB/c mice were purchased from Envigo (an Inotiv company, Chicago, IL, USA) and approved by local authorities (Generalitat de Catalunya, project number 11157) and Universitat Autònoma de Barcelona Ethics Commitee. The animal experiments strictly conformed to the animal welfare legislation of the Generalitat de Catalunya. All the experiments were approved by the local Research Ethics Committee (Procedure 43.19, Hospital de la Vall d’Hebron, Universitat Autònoma de Barcelona).

## 3. Results

### 3.1. Design of L1:P18I10 and L1:T20 Immunogens and Evaluation of HPV:HIV Protein Expression by Using 293F Expression System

**Figure 1:**
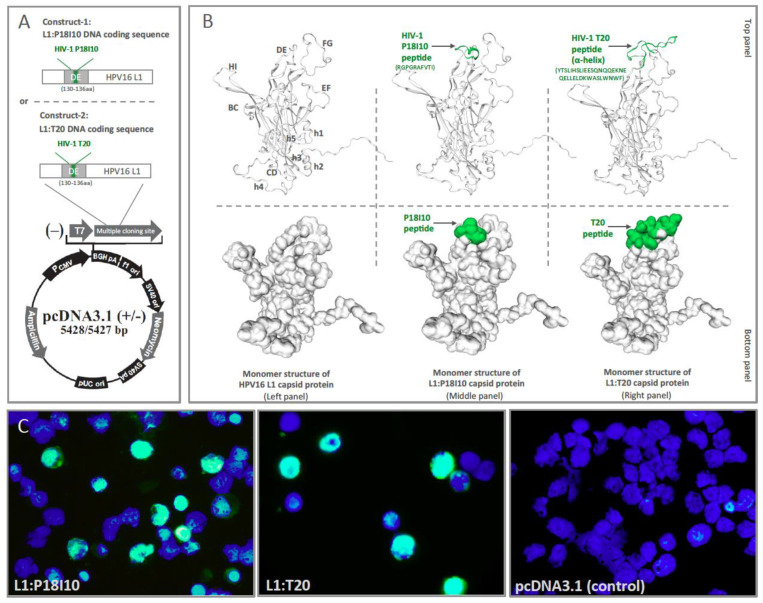
L1:P18I10 and L1:T20 immunogen design and construction of chimeric HPV:HIV VLPs by using 293F expression system. (**A**) The chimeric L1:P18I10 and L1:T20 DNA coding sequence were cloned into pcDNA3.1+ expression vector, respectively, for transient transfection in 293F cells. (**B**) Prediction of monomer structures of HPV16 L1 and chimeric HPV:HIV capsid proteins by using the SWISS-model server. HPV16 major capsid protein L1 (7cn2.1.R) was selected as the structural template to build the HPV16 L1 (left panel), L1:P18I10 (middle panel) and L1:T20 (right panel) capsid protein homology modeling. Secondary structural elements of HPV16 L1 capsid protein are labeled, with letters h1–h5 for the 5 α-helices. Loops of HPV16 L1 capsid protein between strands are labeled BC, CD, DE, EF, FG and HI. Secondary structural elements of HIV-1 P18I10 and T20 peptides L1 are labeled by the arrows (top panel). The part of the P18I10 and T20 peptide that protrudes above the surface of the HPV16 L1 capsid protein are indicated by the arrows (bottom panel). (**C**) Immunofluorescence staining of L1 protein in 293F cells. The following pDNA.L1:P18I10 (left), pDNA.L1:T20 (middle) and pDNA.without insertion (right) were transfected in 293F cells. The transfected cells were probed with anti-HPV16 L1 mAb and detected with anti-mouse IgG-FITC (green channel). Cell nuclei were stained with DAPI (blue channel). Immunofluorescence images were merged by using Adobe Photoshop.

The P18I10 peptide from HIV-1 Env third variable domain (V3) loop and T20 peptide from HIV-1 Env membrane‘s proximal external region (MPER) were inserted into to HPV16 L1 DE loop protein to generate chimeric L1:P18I10 and L1:T20 immunogens. The chimeric L1:P18I10 and L1:T20 DNA coding sequence were cloned into pcDNA3.1 (+) plasmid DNA expression vector for transient transfection in 293F cells (Figure 1A). The monomer structures of HPV16 L1, chimeric L1:P18I10 and L1:T20 capsid proteins were preliminarily predicted using the SWISS-model server (Figure 1B). HPV16 major capsid protein L1 (7cn2.1.R) was selected as the structural template to build the HPV16 L1, L1:P18I10 and L1:T20 capsid protein homology modeling. Compared with the conformation of the bound P18I10 peptide in the previous study [41], the N to C terminus main chain direction of P18I10 peptide on our chimeric L1:P18I10 protein runs right to left (Figure 1B, middle and top panel), and the exposing P18I10 side chains could potentially interact with T cell receptors (Figure 1B, middle and bottom panel). Compared with the structure of HIV-1 fusion inhibitor peptide in the previous review paper [42], the inserted T20 peptide on HPV16 L1 capsid protein is presented as a α-helix-like formation (Figure 1B, right and top panel), and the exposing T20 side chains could potentially recognized by B cell receptors (Figure 1B, right and bottom panel). Since HPV16 L1 capsid proteins could homogeneously assemble into a T = 7 icosahedral particle with 72 pentameric capsomeres [43], the high density display of P18I10 or T20 peptides to the exterior surface of chimeric HPV:HIV VLPs is potentially highly immunostimulatory to induce epitope-specific immune responses.

Since HPV16 L1 protein C terminal sequence mediates cellular nuclear import machinery during infection [44], nuclear localization signals (NLS) of HPV16 L1 protein has been identified in prior studies [45]. The CAMVIR-1 monoclonal antibody was selected to recognize HPV16 L1 epitope (GFGAMDF, 230–236 aa) [39], and fluorescein-based dye FITC was used as reporter to monitor expression of L1:P18I10 and L1:T20 proteins. Immunofluorescence images clearly showed that HPV16 L1 (in green) was mainly localized in the nuclei (in blue) of 293F cells. No L1 signal was observed in control plasmid-transected 293F cells (pcDNA3.1 plasmid without insert) (Figure 1C). The results suggested that both chimeric L1:P18I10 and L1:T20 capsid proteins could be expressed by using polyethylenimine (PEI)-mediated transfection and recognized by HPV16 L1 CAMVIR-1 monoclonal antibody.

### 3.2. Purification of L1:P18I10 and L1:T20 VLPs by Using Chromatographic Methods

**Figure 2:**
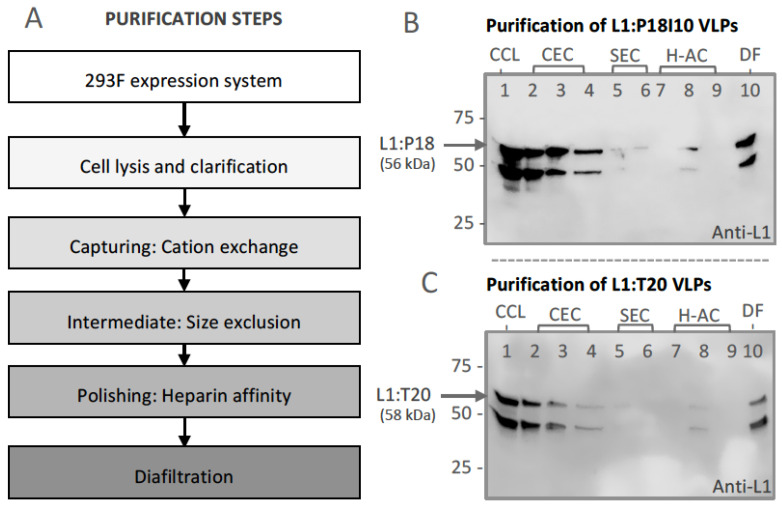
Purification and characterization of L1:P18I10 and L1:T20 VLPs. (**A**) Schematic process flowchart of L1:P18I10 and L1:T20 VLP purification by chromatography. (**B**,**C**) Western blot analysis of L1:P18I10 and L1:T20 VLP samples from each purification step. The signal of L1 in each purification step was characterized by Western blot analysis probed with anti-HPV16 L1 mAb. The arrow indicates the molecular weight ~56 kDa of L1:P18I10 and ~58 kDa of L1:T20 proteins. Lane 1: clarified cell lysate (CCL); Lane 2: flow-through (FT) from cation exchange chromatography (CEC) sample loading; Lane 3: CEC eluate; Lane 4: FT from CEC 2M NaCl regeneration step; Lane 5: size exclusion chromatography (SEC) FT-1; Lane 6: SEC FT-2; Lane 7: FT from heparin affinity chromatography (H-AC) sample loading; Lane 8: H-AC eluate; Lane 9: FT from H-AC 2M NaCl regeneration step; Lane 10: 10-fold diafiltration.

The chimeric L1:P18I10 and L1:T20 proteins were produced by using 293F expression system. The capture, intermediate purification, polishing (CiPP) strategy to develop our chromatographic purification protocol is shown in Figure 2A. Flowthrough (FT) in each purification step were collected and the level of L1 protein expression was detected by Western blot analysis using anti-L1 mAb to trace intermediate HPV:HIV VLPs (Figure 2B,C). A cation exchange (CEC) column was selected as capturing step to isolate HPV:HIV VLPs from host cell proteins (HCPs). The result of CEC FT revealed that most of L1:P18I10 and L1T20 VLPs were lost in the FT over the CEC column (Figure 2B,C, lane 2). Traditional CEC matrices we used heavily rely on diffusion-limited mass transfer [46]. Large macro-molecular complexes, such as our HPV:HIV VLP samples, might be inefficient for the VLP binding to CEC matrices. In order to reach the maximum binding capacities of the CEC column (~50 mg protein/mL resin), we loaded double amount of soluble cell lysate containing approximately 2% of HPV:HIV VLPs into the CEC column. In intermediate purification step, HPV:HIV VLPs were purified using a layered-bead size exclusion chromatography (SEC) resin [38]. Large HPV:HIV particles (>700 kDa) were eluted while most of small impurities were trapped in the beads (Figure 2B,C, lane 5). Due to heparin having a similar structure as DNA and possibly binding to positively charged peptides of conformational HPV16 L1 VLPs, we selected a heparin affinity chromatography (H-AC) as polishing step to remove heterogeneous or closely-related particles [47]. Analysis of densitometry from Western blot analysis and bovine serum albumin (BCA) assay confirmed that purity of L1:P18I10 and L1:T20 VLPs after diafiltration step was high, over 76% (Figure 2B,C, lane 10). The purified L1:P18I10 and L1:T20 VLPs were detected as a band in size of approximately 56 kDa and 58 kDa, respectively. We found that the commercial HPV16 L1 protein and our purified chimeric HPV:HIV VLPs (L1:P18I10 and L1:T20) share the similar protein pattern (a target band >50 kDa and a heterologous lower band <50 kDa). These heterologous lower bands could be detected, especially, when we loaded SDS-PAGE gel with high amount of chimeric HPV:HIV VLP samples (Figure 2B,C, lane 1 to 10). It probably caused by proteolytic degradation or heterogeneous formation of L1 proteins. The similar pattern (2 bands) was also found in many previous HPV16 L1 purification studies [23,48,49,50,51]. These data demonstrated that 293F expression system and chromatographic purification methods are feasible approaches to engineer chimeric HPV:HIV VLPs.

### 3.3. In Vitro Stability and Self-Assembly of L1:P18I10 and L1:T20 VLPs

**Figure 3:**
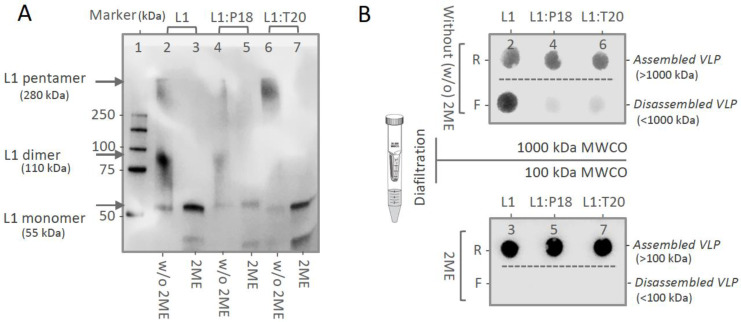
In vitro stability of L1:P18I10 and L1:T20 VLPs. (**A**) Disulfide cross-linking of L1:P18I10 and L1:T20 VLPs in non-reducing SDS-PAGE. The HPV16 L1 VLPs, purified L1:P18I10 and L1:T20 VLPs were mixed with Laemmli sample buffer in the absence or presence of 2-mercaptoethanol (2-ME), respectively, and analyzed by non-reducing SDS-PAGE. The position of the L1 monomer (55 kDa), L1 dimer (110 kDa) and L1 pentamer (280 kDa) are indicated by the arrow. Lane 1: protein molecular weight marker; Lane 2: HPV16 L1 VLP; Lane 3: HPV16 L1 VLP treated with 2-ME; Lane 4: L1:P18I10 VLP; Lane 5: L1:P18I10 VLP treated with 2-ME; Lane 6: L1:T20 VLP; Lane 7: L1:T20 VLP treated with 2-ME. (**B**) Molecular mass analysis of L1:P18I10 and L1:T20 VLPs. Assembled VLPs un-treated with 2-ME (lane 2, 4 and 6) were filtered out through 1000kDa molecular weight cutoff (MWCO) diafiltration devices (upper panel). Disassembled VLPs treated with 2-ME (lane 3, 5 and 7) were filtered out through 100kDa MWCO centrifugal filter devices (lower panel). Retentates (R) were collected from filter device sample reservoirs, while the filtrates (F) were collected at the bottom of centrifuge tubes. The L1 protein signal was detected by using dot blot probed with anti-HPV16 L1 mAb.

In order to confirm that purified HPV:HIV VLPs displayed similar in vitro stability to HPV16 L1 VLPs, we performed non-reducing SDS-PAGE to evaluate disulfide cross-linking of HPV:HIV capsid proteins (Figure 3A). It is known that pH, ionic strength, temperature [52] and redox environment all correlate with disulfide bonds of HPV16 L1 capsid proteins [53]. HPV L1 VLPs tend to self-assemble at low pH and high ionic strength. Maximal disassembly of VLPs typically require exposure to a high concentration of reducing agent, such as 5% 2-mercaptoethanol (2-ME) for a relatively long duration [54]. In the absence of reducing agents 2-ME, only a small portion of the HPV-16 L1, L1:P18I10 and L1:T20 protein migrated to monomers with an apparent molecular weight (MW) of 55 kDa. Approximately 70% of L1 proteins were disulfide bonded into larger dimers or pentamers, with predicted MW of 110 kDa and 280 kDa (Figure 3A, lane 2, 4 and 6). By contrast, almost all of HPV-16 L1, L1:P18I10 and L1:T20 proteins in the disassembly buffer appeared monomeric structure in non-reducing SDS-PAGE (Figure 3A, lane 3, 5 and 7). These results indicated that in vitro stability of purified L1:P18I10 and L1:T20 VLPs presented similar disulfide cross-linking pattern as HPV16 L1 VLPs under the same pH, ionic strength and thermal conditions. The purified VLPs appear to be broken down to the level of pentarmers, trimers and dimers following long-term exposure to high concentrations of reducing agent. These data are in concordance with previous studies [53,54] However, disassembly of HPV:HIV VLPs was still far from complete (monomers).

To demonstrate that purified HPV:HIV proteins by chromatography are able to self-assemble to icosahedral particles, we further performed molecular mass analysis under the same reducing condition (5% 2-ME). The commercial HPV16 L1, purified L1:P18I10 and L1T20 proteins without reducing agent treatment were filtered out through 1000 kDa molecular weight cut-off (MWCO) diafiltration devices individually (Figure 3B, top panel). The L1 monomers (55 kDa), oligomers (110~200 kDa) or pentameric capsomers (280 kDa) were expected to pass through an ultrafiltration membrane retaining the integral VLPs (MW~20,000 kDa). The L1 signal of commercial HPV16 L1 proteins was detected in both retentates and filtrates. Most of purified L1:P18I10 and L1T20 proteins formed large particles (>1000 kDa) and were preserved in retentates. The pattern was in coincidence to the data that were observed in non-reducing SDS-PAGE. Although all the VLP groups treated with 2-ME were showed in a monomeric band (~55 kDa) in the non-reducing SDS-PAGE (Figure 3A, lane 3, 5 and 7). However, the corresponding reduced VLPs were not filtered out through 100kDa ultrafiltration membranes (Figure 3B, bottom panel). These results suggested that chimeric HPV:HIV proteins were capable of self-assembling to larger particles, but maximal disassembly of VLPs into monomers required not only the reduction of disulfide bonds but also other denaturing factors, such as pH or ionic strength.

### 3.4. Morphological Characterization of L1:P18I10 and L1:T20 VLPs

**Figure 4:**
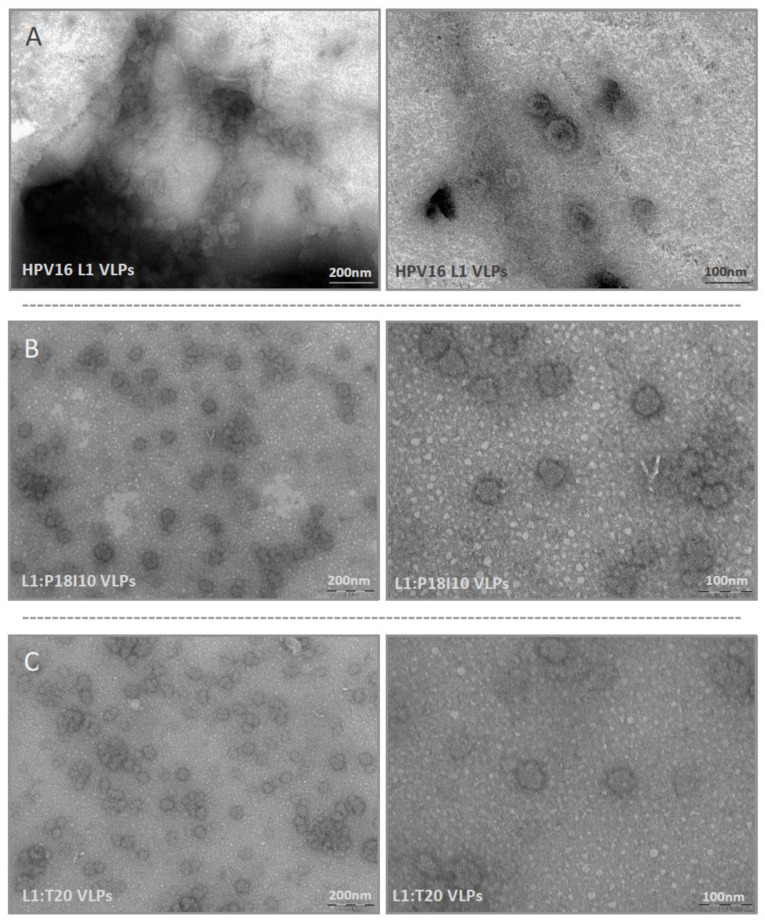
Electron micrographs of L1:P18I10 and L1:T20 VLPs. (**A**) Morphology of HPV16 VLPs. (**B**) Morphology of L1:P18I10 VLPs. (**C**) Morphology of L1:T20 VLPs. Purified VLPs were absorbed on UV-charged carbon-coated copper grids, and negatively stained with 2% uranyl acetate. Images were acquired under transmission electron microscopy. The bar represents 200 nm at magnification 59,000 (left panel) and 100 nm at magnification 135K (right panels), respectively.

Transmission electron microscopy (TEM) was used to examine morphologic conformation of HPV16 L1 and HPV:HIV VLPs. The HIV-1 P18I10 and T20 peptides were inserted into DE loops of HPV16 L1 protein, respectively. These commercial HPV16 L1 and chimeric HPV:HIV capsid proteins can spontaneously self-assemble in vitro into integral VLPs in a diameter of approximately 50–60 nm (Figure 4A–C, right panel). Compared to electron micrographs of HPV16 L1 VLPs published in previous studies [55,56], the icosahedral structure of HPV16 L1, L1:P18I10 and L1:T20 VLPs here were less contrast and vague. It could be attributed to the negative stain reagent that we used in this study. In our recent study published in previous study [37] and another ongoing paper under peer review (data not shown), we have got good quality and resolution of electron micrographs when yeast- and baculovirus-derived L1:P18I10 VLPs (the same chimeric construct) were equilibrated in PBS and negative-stained with phosphotungstic acid (PTA). Because we used the different VLP production and purification system in this study, mammalian cell-derived L1:P18I10 and L1:T20 VLPs were equilibrated with Tris-HCl and negative-stained with uranyl acetate. Although the electron micrographs could be improved, we could still observe a clear pattern that most of the HPV16 L1, L1:P18I10 and L1:T20 capsid protein could self-assemble into morphological VLPs under TEM screen (Figure 4A–C, left panel). Basically, HPV VLPs are protected against aggregation in high salt conditions [57]. Some of detectable aggregation of HPV16 L1 and HPV:HIV VLPs in low salt Tris-HCl buffer could be seen under the lower magnification (Figure 4A–C, left panel). From these results, we concluded that modification of partial L1 DE loop sequence by insertion of HIV-1 P18I10 or T20 peptides did not significantly affect the morphology of HPV:HIV VLPs.

### 3.5. Presentation and Reactivity of the HPV-16 and HIV-1 Epitopes

**Figure 5:**
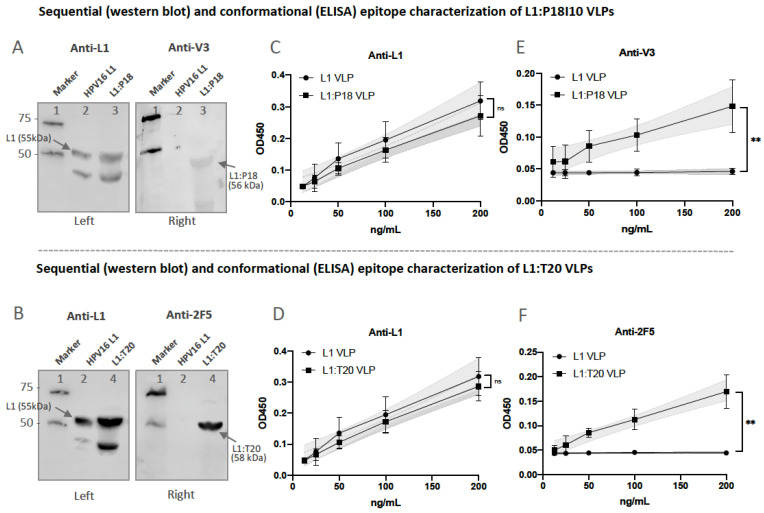
Presentation of HPV-16 and HIV-1 epitopes. (**A**,**B**) Sequential epitope detection of chimeric L1:P18I10 and L1:T20 VLPs. The purified L1:P18I10 and L1:T20 VLPs were analyzed by Western blot, using anti-HPV16 L1, anti-HIV-1 gp120 V3 and 2F5 mAb. The HPV16 L1 VLPs were used as a control. The molecular weight of the L1 (55 kDa), L1:P18I10 (56 kDa) and L1:T20 (58 kDa) proteins are indicated by the arrow. Lane 1: protein molecular weight marker; Lane 2: HPV16 L1 protein; Lane 3: L1:P18I10 protein; Lane 4: L1:T20 protein. (**C**,**D**) Binding of HPV16 L1 mAb to chimeric L1:P18I10 and L1:T20 VLPs. (**E**) Binding of anti-HIV-1 gp120 V3 *mAb to chimeric L1:P18I10 VLPs.* (**F**) Binding of HIV-1 2F5 mAb to chimeric L1:T20 VLPs. The line graph of indirect ELISAs were performed to detect the conformational epitopes of recombinant HPV16 L1, L1:P18I10 and L1:T20 VLPs bound to anti-L1, anti-HIV-1 gp120 V3 or 2F5 mAbs, respectively. Data are representative of three independent experiments. Simple linear regression test was done to compare the line difference of purified L1:P18I10 and L1:T20 VLPs with the standard curve of commercial L1 VLPs; ns: not significant; ** *p* < 0.01.

To confirm that sequential HIV-1 P18I10 or 2F5 epitopes were presented in chromatography-purified L1:P18I10 or L1:T20 VLPs, Western blot analysis and indirect ELISA were preformed using epitope-specific mAbs. We selected a well-known monoclonal antibody (mAb), designated CAMVIR-1, to recognize the highly conserved epitope (GFGAMDF, aa 230–236) of HPV16 L1 protein [39,58]. A previously published mAb targeting HIV-1 gp120 V3 loop epitope (RIQRGPGRAFVTIGK, aa308–322) was chose to detect sequential P18I10 epitopes (RGPGRAFVTI, aa311–320) [59]. On the other hand, the broad neutralizing antibody (bnAb) recognizing HIV-1 gp41 2F5 epitope (ELDKWA) against a broad variety of laboratory HIV-1 strains was chose for T20 peptide characterization [60,61]. Western blot assay probed with HPV16 L1 mAb showed bands 55, 56 and 58 kDa corresponding to HPV16 L1, L1:P18I10 and L1:T20 protein, respectively (Figure 5A,B, left). The molecular weight (MW) of L1:P18I10 protein was similar to HPV16 L1 protein (Figure 5A, left). The band corresponding to L1:T20 protein was observed slightly higher than HPV16 L1 protein, as predicted from the additional amino acid sequence (Figure 5B, left). The bands of approximately 56 and 58 kDa, corresponding to L1:P18I10 and L1:T20 protein, were detected in Western blot assay probed with anti-HIV-1 gp120 V3 and 2F5 mAb, respectively (Figure 5A,B, right). We thought the molecular weight (MW) of anti-2F5-stained L1:T20 protein is correct and fitted the expected 58 kDa (Figure 5B, right). However, the MW of anti-HIV-1 gp120 V3-stained L1:P18I10 protein is a bit lower (Figure 5A, right). This could be attributed to the heterogeneous structure of chimeric L1:P18I10 proteins. Since the sequential epitope conformation might be lost under the denaturing condition in SDS-PAGE. Therefore, we further performed indirect ELISA to demonstrated the conformational P18I10 peptide properly presented on our chimeric L1:P18I10 VLPs (Figure 5E). On the other hand, anti-HIV-1 gp120 V3 loop antibody that we used recognized whole HIV-1 V3 loop rather than P18I10 epitope. Consequently, the overall anti-V3 signal was lower (Figure 5A,B, right panels). Even so, these results indicated that the sequential HIV-1 P18I10 and T20 peptides are presented in the HPV:HIV VLPs.

Moreover, to determine whether HIV-1 conformational epitopes presented on HPV:HIV VLPs could be identified by the gp120 V3 and 2F5 neutralizing antibodies in vitro, we performed indirect ELISA assay to check the epitope-binding specificity and reactivity. As shown in Figure 5C,D, HPV16 L1, L1:P18I10 and L1:T20 VLPs were recognized by anti-L1 mAb. We performed linear regression analysis to compare the slope of each dilution line. The results revealed that L1 epitope-binding specificity of either L1:P18I10 or L1:T20 VLPs was not different from HPV16 L1 VLPs. Moreover, anti-HIV-1 gp120 V3 mAb was able to bind L1:P18I10 VLPs, but not HPV16 L1 VLPs (Figure 5E). In addition, the 2F5 mAb could recognize L1:T20 VLPs, but not HPV16 L1 VLPs (Figure 5F). After linear regression analysis, the difference of gp120 V3 epitope-binding specificity between L1:P18I10 and HPV16 L1 was extremely significant (*p* < 0.01%). The 2F5 epitope-binding specificity of L1:T20 VLPs was significantly different from HPV16 L1 VLPs (*p* < 0.01%). These pattern revealed that hydrophobic cellular lipids were not necessary for the binding of 2F5 neutralizing antibodies to HPV:HIV VLPs in vitro. Although the reactivity of anti-HIV-1 gp120 V3 and 2F5 neutralizing antibodies to HPV:HIV VLPs is relatively mild, the binding of anti-HIV-1 gp120 V3 and 2F5 mAb to HPV:HIV VLPs were significantly epitope-specific.

### 3.6. Immunogenicity of L1:P18I10 and L1:T20 VLPs after BALB/c Mice Immunization

**Figure 6:**
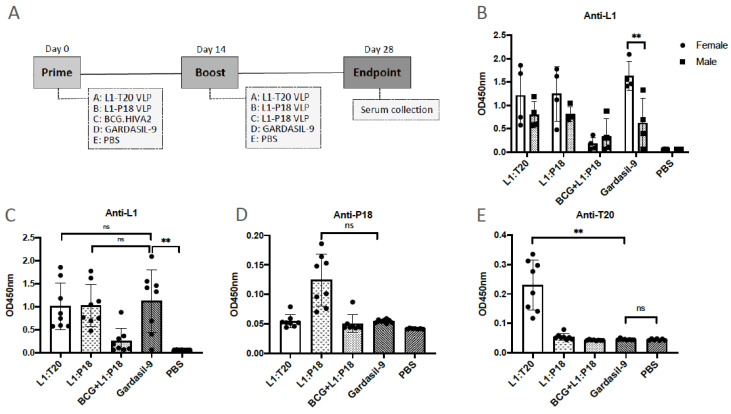
Induction of humoral immune responses by L1:P18I10 and L1:T20 VLPs in BALB/c mice. The immunization schedule is depicted in (**A**) All mouse groups had equal gender distribution (male *n* = 4 and female *n* = 4). Groups A and B: homologous prime-boost immunization with 10 μg L1:P18I10 or L1:T20 VLPs intramuscularly (i.m.); Group C: priming with 10^6^ cfu rBCG.HIVA intradermally (i.d.) and boosting with 10 μg L1:P18I10 VLPs i.m.; Group D: homologous prime-boost vaccination with Gardasil-9 containing 10 μg of HPV16 L1 VLPs i.m.; Group E: immunization twice with PBS buffer. The prime-boost interval was 2 weeks. The end point of this trial was on day 28. Sera were collected and diluted at a titer of 1:50 for ELISA assay. (**B**) HPV L1-specific IgG in male and female mice. (**C**–**E**) Epitope-specific IgG induced by L1:P18I10 and L1:T20 VLPs. ELISA was performed to analyze anti-L1, anti-P18I10 and anti-T20 IgG induced by BALB/c mice following different prime-boost combinations as described above. Data are shown as mean ± S.D. One-way ANOVA (nonparametric) test was done to compare differences between groups. OD: opticaldensity. Ns not significant; ** *p* < 0.01.

We evaluated the HPV16- and HIV-1-specific immune responses after BALB/c mice immunization with L1:P18I10 and L1:T20 VLPs. The immunization schedule is shown in Figure 6A. Because VLP-induced immunogenicity following mucosal administration was generally weaker than following systemic administration, mice were immunized intramuscularly with one sixth of the Gardasil-9 HPV16 L1 dose [22,62]. The aluminum hydroxy phosphate sulfate adjuvant for a dose (10 μg/100 μL) of chimeric HPV:HIV VLPs was adjusted to the same concentration (1 mg/1 mL) as Gardasil-9. In order to assess the sex difference in the outcomes of vaccination, a comparison of antibody responses between male (*n* = 4) and female (*n* = 4) mice were evaluated. In the group of L1:P18I10 VLP, L1:T20 VLP and Gardasil-9, anti-HPV16 L1 antibody responses of female mice were on average higher than male mice (Figure 6B). 2 out of 4 (50%) L1:P18I10 VLP-immunized females, 3 out of 4 (75%) L1:T20 VLP-immunized females and all (100%) Gardasil-immunized female mice elicited higher titer of anti-L1 antibodies than male mice. Anti-L1 responses induced by female mice in Gardasil-9 group was significantly higher than male mice (*p* = 0.0041) (Figure 6B). A very low level of anti-L1 antibody responses were detected in the group of rBCG.HIVA priming and L1:P18I10 VLP boosting. This pattern corresponds with previous findings describing that BCG predominantly induces T-cell responses rather than IgG production [63].

We assessed if mice immunized with L1:P18I10 and L1:T20 VLPs could induce HPV-16 L1-specific and HIV-1 epitope-specific antibodies in BALB/c mice. VLP-induced IgG antibodies in murine sera were measured by ELISA coated with recombinant HPV16 L1 protein, P18I10 or T20 peptides, respectively. A statistically difference in L1-specific IgG at a serum titer of 1:50 was detected in Gardasil-9 group in comparison with PBS control group (*p* = 0.0039). The anti-L1 responses among Gardasil-9, L1:P18I10 and L1:T20 VLP-immunized mice were similar and did not differ significantly (Figure 6C). The BCG.HIVA^2auxo.int^ prime and L1:P18I10 VLP boost mice elicited a very low level of anti-L1 IgG. These results suggested that HPV:HIV VLP-immunized mice produced the same level of anti-L1 IgG as Gardasil-9-immunized mice (Figure 6C). Although L1:P18I10 VLP group numerically appear to a trend toward higher level of P18I10 epitope-specific IgG than other immunization groups, these differences were not statistically significant (Figure 6D). In some of L1:P18I10 VLP-immunized mice (4 out of 8, 50%), higher anti-P18I10 binding antibodies were observed compared to Gardasil-9-immunized mice. Alternatively, a T-test analysis revealed that the difference between L1:P18I10 VLP and Gardasil-9 group was significant (*p* = 0.005) (data not shown). In L1:T20 VLP group, a significantly higher antibody response against T20 peptide was detected compared to Gardasil-9 group (*p* = 0.0083). As expected, anti-T20 titers were undetectable in Gardasil-9, PBS, L1:P18I10 VLP and rBCG.HIVA prime combined with L1:P18I10 VLP boost groups (Figure 6E). The titer of T20 peptide-specific antibody was relatively low. This is likely due to: (1) T20 is subdominant peptide; (2) elicitation of MPER or 2F5 neutralizing antibodies requires peptide-lipid conjugates (Figure 6E). Overall, our results demonstrated that L1:P18I10 and L1:T20 VLPs could induce HPV16- but weak HIV-1-specific antibody responses in mice.

**Figure 7:**
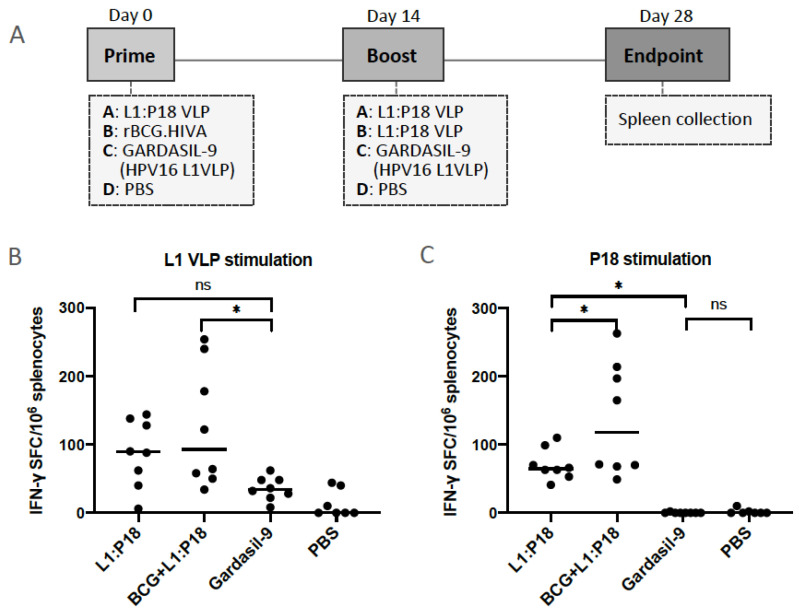
Induction of HPV16 and HIV-1 specific T cell responses by chimeric L1:P8I10 VLPs and rBCG.HIVA in BALB/c mice. The immunization schedule is depicted in (**A**). All mouse groups had equal gender distribution (male *n* = 4 and female *n* = 4). Group A: homologous prime-boost immunization with 10 μg L1:P18I10 VLPs intramuscularly (i.m.); Group B: priming with 106 cfu rBCG.HIVA intradermally (i.d.) and boosting with 10 μg L1:P18I10 VLPs i.m.; Group C: homologous prime-boost vaccination with Gardasil-9 containing 10 μg of HPV16 L1 VLPs i.m.; Group D: immunization twice with PBS buffer. The prime-boost interval was 2 weeks. The end point of this trial was on day 28. Splenocytes were isolated for IFN-γ ELISpot assay. T-cell immune responses to HPV16 and HIV-1 were assessed ex vivo by IFN-γ ELISpot after splenocyte stimulation with HPV16 L1 VLP and P18I10 peptide. (**B**,**C**) HPV16 L1- and HIV-1 P8I10-specific T-cell responses elicited by L1:P8I10 VLPs and rBCG.HIVA prime combined with L1:P18I10 VLP boost. Data are shown as median ± S.D. One-way ANOVA test was done to compare differences between groups. Ns not significant; * *p* < 0.05.

To evaluate the HPV and HIV-specific T-cell immune responses in mice, we followed the immunization schedule shown in Figure 7A. In addition, heterologous BCG.HIVA^2auxo.int^ priming and L1:P18I10 VLPs boosting was compared with homologous L1:P18I10 VLP prime and boost immunization to evaluate the frequency of specific-HPV16 and HIV-1 T-cell immune responses. There were no differences between Gardasil-9 and PBS groups regarding the IFN-γ secretion after splenocytes stimulation with HPV16 L1 VLPs. Differences in L1-specific IFN-γ secretion were also not significant between L1:P18I10 VLP and Gardasil-9 groups. We deduced that the weak L1-specific IFN-γ secretion might be attribute to the low concentration (2 μg/mL) of HPV16 L1 VLPs used as stimuli. The group of BCG.HIVA^2auxo.int^ prime and L1:P18I10 VLP boost induced higher frequency of IFN-γ secreting splenocytes and significant difference in IFN-γ secretion were observed when compared with mice vaccinated with Gardasil-9 group (*p* = 0.0103). 3 out of 8 mice (~38%) elicited the highest L1-specific IFN-γ responses (Figure 7B). This might be attributed to the unspecific adjuvanticity of BCG according our previous studies [64,65,66]. The evident priming effect, even by wild-type BCG, is in line with the ability of rBCG derivatives to act as potent adjuvants for subsequent boosting vaccines [64,65]. Regarding the HIV-1-specific T-cell responses, the highest total magnitude of IFN-γ spot-forming cells (SFC)/10^6^ splenocytes was observed in BCG.HIVA^2auxo.int^ primed mice compared to mice receiving Gardasil-9 vaccines or L1:P18I10 VLPs. Mice primed with BCG.HIVA and boosted with L1:P18I10 VLPs elicited significantly higher IFN-γ secretion compared with mice vaccinated with L1:P18I10 VLP homologous prime-boost (*p* = 0.0268). In addition, a significantly higher IFN-γ secretion was observed in mice vaccinated with L1:P18I10 VLPs in comparison with mice receiving Gardasil-9 vaccines (*p* = 0.0157) (Figure 7C). As expected, the IFN-γ secretion was undetectable in Gardasil-9 and PBS groups. These results demonstrated that (1) L1:P18I10 VLPs elicited HIV-1-specific T-cell immune responses; (2) L1:P18I10 VLPs elicited HPV-specific T-cell immune responses and (3) BCG.HIVA^2auxo.int^ could boost the HIV-1-specific T-cell immune responses elicited by L1:P18I10 VLP.

## 4. Discussion

Both HPV16 and HIV-1 are sexually transmitted diseases and are currently the focus of many vaccine studies. Although HPV prophylactic vaccines have been commercialized and HIV-1 transmission has been greatly controlled by anti-retroviral treatment (ART) and PrEP, an effective, safe and affordable chimeric HPV:HIV vaccine against both viruses is an urgent need. In this study, (1) we demonstrated that the 293F expression system and the chromatographic purification method could be feasible approaches to produce and purify chimeric L1:P18I10 and L1:T20 VLPs; (2) we confirmed that the insertion of P18I10 or T20 peptides into the DE loop of HPV16 L1 capsid proteins did not affect in vitro stability, self-assembly and morphology of chimeric HPV:HIV VLPs; (3) The sequential and conformational P18I10 or T20 peptides exposed to DE loops of chimeric HPV:HIV VLPs could be detected by anti-HIV-1 gp120 V3 and 2F5 neutralizing antibodies in vitro; (4) The chimeric L1:P18I10 and L1:T20 VLPs could elicit HPV but weak HIV-1-specific binding antibodies in BALB/c mice. Furthermore, the insertion of HIV-1 P18I10 or T20 peptides into HPV16 L1 protein did not affect HPV16 L1-specific antibody induction in vivo; (5) L1:P18I10 VLPs could induce both HPV16 and HIV-1-specific T-cell responses; (6) BCG.HIVA prime and L1:P18I10 VLP boost elicited highest magnitude of IFN-γ producing splenocytes in comparison with L1:P18I10 VLPs homologous prime-boost in BALB/c mice. These finding supported further development of HIV-1 vaccines based on rBCG and chimeric HPV:HIV VLPs. All in all, this study provides a baseline strategy that may be worthy to support the global efforts to develop novel chimeric VLP-based vaccines for controlling HPV and HIV infections.

In this study, L1:P18I10 and L1:T20 VLPs could induce the same level of anti-HPV16 L1 binding antibodies as HPV Gardasil-9 vaccine. However, L1:P18I10 and L1:T20 VLPs only elicit low level of P18I10 and T20 epitope-specific HIV binding antibody responses. We hypothesized that might be due because ELISA assay plates were coated with recombinant HPV16 L1 protein, HIV-1 P18I10 peptide and HIV-1 T20 peptide, respectively. The murine anti-L1 antibodies induced by our L1:P18I10 and L1:T20 VLPs might target multiple binding epitopes of L1 protein. By contrast, murine anti-HIV-1 antibodies elicited by our L1:P18I10 and L1:T20 VLPs only target single HIV peptide (P18I10 or T20) on HPV:HIV VLPs. Consequently, the overall maximum OD450 values of anti-HIV-1 binding antibodies were much lower than anti-HPV16 L1 antibodies.

The reason that we selected HPV16 L1 capsid protein DE loop as preliminary insertion region of HIV-1 immunogen referred to a previous study that immunized mice with bovine papillomavirus (BPV):HIV VLPs for inducing antibody responses [20]. It is known that the epitopes located within surface-exposed DE and FG loops of the HPV L1 major capsid proteins dominantly contribute to the vaccine-induced cross-neutralizing antibodies [67]. Furthermore, it has been shown by previous studies that insertion of HIV-1 MPER into BPV L1 DE loop could induce partially neutralizing antibodies that specifically recognized the native conformation of MPER in HIV-1 Env [20]. However, no data has shown whether the recombinant BPV L1:MPER VLP affects the immunogenicity and neutralization against BPV after insertion of HIV-1 MPER into BPV L1 protein. In our study using HPV16 L1 VLPs as HIV antigen delivery vector, we have observed that HIV-1 peptide insertion has not modified HPV L1 structure, and that the chimeric HPV:HIV proteins still holds the capacity to form VLPs. In addition, we carried out the concept of immunobridging to demonstrate comparable immune responses between a HPV:HIV VLP candidates (ours) and an approved VLP based HPV vaccine (licensed Gardasil-9). Our data revealed that the insertion of HIV-1 P18I10 or T20 peptide into chimeric HPV:HIV VLPs could elicit the similar titer of HPV16 L1-specific binding antibodies, compared to HPV Gardasil-9 vaccine. The type-specific anti-HPV16 L1 binding antibodies observed with licensed HPV Gardasil-9 VLP vaccine compared with chimeric versions of HPV Gardasil-9 VLP vaccine was in concordance with our data.

The length and site of optimal HIV-1 foreign antigen incorporated into the HPV16 L1 VLPs and the in vitro stability of the resulting chimeric HPV:HIV VLPs should be verified before mice immunization. Our current study have shown that the insertion of P18I10 or T20 peptides into HPV16 L1 protein did not affect in vitro stability, self-assembly and morphology of chimeric HPV:HIV VLPs. These results were in concordance with previous studies indicating that insertion of HIV-1 MPER domain into BPV L1 DE loop sequence did not influence the capacity of BPV L1 capsid protein self-assemble to VLPs [20]. Basically, epitopes located within surface-exposed DE and FG loops of the HPV L1 capsid proteins dominantly contribute to induce L1-specific cross-neutralizing antibodies [67]. Here, we demonstrated that insertion of HIV-1 P18I10 or T20 peptides into HPV16 L1 DE loop did not affect L1-specific antibody induction by chimeric HPV:HIV VLPs after mice immunization. In addition, the HIV-1 P18I10 or T20 epitopes onto HPV16 DE loops of chimeric HPV:HIV VLPs were detected in vitro and were immunogenic in vivo. HPV16 L1 VLPs constitute a potential scaffold for surface display of the HIV-1 epitope of interest. In this study, we have performed indirect ELISA to demonstrated the conformational P18I10 and T20 peptide presented on the surface of our chimeric HPV:HIV VLPs. In the future, the immune-electron microscopy could also be an additional approach to demonstrate P18I10 or T20 antigen structural localization and organization within HPV:HIV VLPs.

Self-assembly is a representative index of in vitro stability of HPV16 L1 VLPs. It is known that pH, ionic strength, temperature [52] and redox environment all correlate with disulfide bonds of HPV16 L1 capsid proteins [53]. The earlier works on papillomavirus VLPs suggested the importance of disulfide bonds to L1 major capsid self-assembly [68,69]. Disulfide formation indicated a higher cysteine of L1 capsid protein in an appropriate geometry [53]. These disulfide cross-links connecting residues 175 and 428 (for HPV16) that stabilize HPV virions and VLPs [70]. In this study, we analyzed intermolecular disulfide cross-linking pattern as an indirect evidence to prove our L1:P18I10 and L1:T20 capsid proteins tend to self-assembly in vitro. In Figure 3A, we demonstrated that purified L1:P18I10 and L1:T20 VLPs presented similar intermolecular disulfide cross-linking pattern as HPV16 L1 VLPs under the same pH, ionic strength and thermal conditions. In Figure 3B, we further demonstrated that purified L1:P18I10 and L1:T20 proteins were capable of self-assembling to larger particles (larger than L1 pentamer MW 280 kDa) in vitro. Therefore, together with the morphological self-assemble VLP pattern (although the icosahedral structure were not quite good) observed in Figure 4, we concluded our purified chimeric HPV:HIV VLPs is stable in vitro. Besides VLP stability, many other critical factors that could affect the immunogenicity of chimeric HPV:HIV VLPs should be considered, such as immunogen insertion site among different loops of VLPs [71], dose, prime-boost intervals, and administration route [22] etc.

The P18I10 peptides derived from HIV-1 gp120 V3 loop are presented in HIV-infected cells by major histocompatibility complex (MHC-I) class I molecules [26]. CD8+, cytotoxic T lymphocytes (CTL), could recognize MHC-I restricted P18I10 antigens and secreting a variety of cytokines, such as IFN-γ to eliminate HIV-infected cells [72,73,74,75]. Recombinant viral or plasmid DNA are good vaccine vehicles to express P18I10 peptides in host cells and induce P18I10-specific cellular responses through MHC-I pathway [76,77,78,79]. For instance, a combined regimen of DNA prime and modified vaccinia virus Ankara (MVA) boost was efficient for the induction of IFN-γ and CTL responses against the P18I10 epitope [79]. On the contrary, exogenous P18I10 peptides are not efficiently presented to CD8+ T-cells by MHC-I pathway [80,81] and require the participation of appropriate adjuvants [82,83,84,85] or antigen carriers, such VLPs. For instance, immunogenicity of synthetic P18I10 peptides supplemented with adjuvants was marginal due to the absence of T-helper determinants [82,83,84,85]. It has been reported previously that HIV-1 Gag VLPs [86], hepatitis B surface antigen (HBsAg) VLPs [87], parvovirus VP2 VLPs [88] and papillomavirus L1 VLPs [17,18,19] could act as delivery vectors for MHC-I-restricted CTL epitope presentation in vivo. Although the mechanism of VLP-induced MHC-I-restricted T-cell responses is still unclear, the particulate structure of VLPs might benefit endocytic uptake of macrophages or dendritic cells, thus accessing the cytosol and subsequently entering typical MHC-I pathway [89,90]. In addition, the MHC-I-restricted P18I10 determinant was observed to induce CD4+ helper T-cell responses itself through an MHC-II pathway [91,92]. Hybrid BPV1 L1 VLPs can be used as antigenic epitope carriers to elicit therapeutic virus-specific CTL responses through MHC-I and MHC-II pathways [17,19], providing a promising strategy for the vaccine design to control viral infection. Some early studies also indicated BPV L1 VLPs expressing HPV16 E7 epitope or P18I10 epitope of HIV-1 gp120 V3 loop induced mucosal surfaces and also systemic VLP epitope-specific humoral and T cell immunity [18]. In line with previous studies, we have preliminarily demonstrated that our chimeric HPV:HIV (L1:P18I10) VLPs could induce HIV-specific T-cell immune responses in BALB/c mice after splenocytes stimulation with P18I10 peptide. In addition, BCG.HIVA priming enhanced the HIV-1-specific T-cell immune responses in mice. However, the multifunctional T-cell immune responses induced by L1:P18I10 VLPs would need further immunological studies.

Broader CD8+ T-cell responses against multiple conserved CTL epitopes are beneficial to overcome HIV-1 genetic diversity and escape [7,93,94,95,96,97,98,99,100]. The rational design of HIV-1 T-cell immunogens, such as HIVA, should have the potential to respond to multiple CTL epitopes [34]. The HIV-1 HIVA immunogen, designed by Dr. Tomas Hanke, is composed of the full-length HIV-1 Gag protein combined with multiple CTL epitopes including P18I10 epitopes at the C-terminus [34]. The DNA, MVA and rBCG were selected as HIVA immunogen delivery vehicles and induced high magnitude and breadth of CTL epitope-specific cellular responses by using heterologous prime-boost regimes in mouse and non-human primate (NHP) models [34,101]. Our prior studies have shown that rBCG.HIVA prime in combination with MVA.HIVA boost elicited HIV-1-specific IFN-γ producing CD8+ T-cells in BALB/c mice [31,32,33,102]. Interestingly, VLPs could be a potential booster to increase HIV-specific cellular responses in the heterologous immunization with rBCG [29,30] or DNA vaccines [103,104]. For example, rBCG expressing HIV-1 Gag protein could effectively prime the T-cell immune system for a boost with a Gag VLPs in NHP models [29,30]. In the current study, we have demonstrated that rBCG.HIVA priming could boost the T-cell immune responses induced by HPV:HIV (L1:P18I10) VLPs. We will further investigate the magnitude of polyfunctional CD4+, CD8+ and memory T-cell responses generated by this rBCG prime and VLP boost regime.

Currently, our research group is focusing on the development of promising rBCG:HIV vaccines expressing novel HIV-1 T-cell immunogens, such as tHIVconsvX and HIVACAT (HTI) T cell immunogen, to improve HIV-1 variant match and T-cell response breadth. The 2nd-generation HIVconsvX immunogens were designed by redefining the group M conserved regions and utilizes a bivalent mosaic design to maximize the match of potential 9-mer T-cell epitopes in the vaccine to global variants [64]. The HTI immunogen was designed to cover T-cell targets against which T-cell responses are predominantly observed in HIV-1-infected individuals with low HIV-1 viral loads [65,66]. Because papilloma VLPs have been proved to be a multiple CTL epitope carrier [17], we aimed to construct chimeric HPV16 L1 VLPs carrying multiple conserved HIV-1 CTL epitopes in combination with rBCG expressing ThivconsvX or HTI T-cell immunogens to induce broader CTL immune responses against HIV-1. The high density and multiple copies of the HIV-1 CTL epitope presented on chimeric HPV:HIV VLPs might improve antigen delivery to the immune system and induce a higher frequency of CTL responses. By contrast, recombinant BCG is more likely to generate a lower frequency of CTL responses due to slow replication in vivo. Because BCG has a distinct influence on the differentiation of T cells, which means that BCG can induce memory CD8+ T cells through the participation of CD4+ T-helper cells [105], this immunogenic property might make BCG suitable as a priming agent in heterologous prime-boost regimens. Thus, we expected that our HPV:HIV VLPs might appear to be a promising booster to increase the magnitude and breadth of HIV-1 CTL responses when priming with a recombinant BCG expressing novel HIV-1 T-cell immunogen.

The T20 peptide contains a highly conserved linear epitope 2F5 (ELDKWA). The 2F5 antibody collected from long-term HIV-infected patients was reported to have broadly neutralizing efficacy [106,107]. The MPER of gp41 is considered to be poor immunogenic, perhaps related to its location close to cellular and viral phospholipid bilayer [108]. Using DNA vectors presenting MPER in a lipid environment is beneficial to induce gp41-specific nAbs [109,110,111]. For instance, the HIV-1 T20-encoding DNA vaccines, designed by Dr. Britta Wahren, have been demonstrated to induce cross-clade neutralizing antibody (nAb) responses [109]. By contrast, many early attempts to induce nAbs targeting gp41 by using peptide or subunit vaccine strategies have failed [112,113,114,115,116]. Recently, some studies indicated BPV L1 VLPs expressing 2F5 epitope or MPER of HIV-1 gp41 induced 2F5-specific antibodies in mice resulted in cross-clade neutralization [20,21]. A similar immunogenicity pattern was found when hepatitis B surface antigen (HBsAg) was fused with HIV-1 2F5 epitope or MPER [59,117,118,119]. Here, we demonstrated that presentation of HIV-1 T20 and P18I10 peptide in our chimeric HPV16 L1 VLP could induce antibody responses against HIV-1 and HPV16. Neutralizing epitopes stabilized on a conformational scaffold, such as HIV-1 functional spikes or VLPs, could be the mainstream of B-cell immunogen design for achieving broad neutralizing antibodies (bnAbs) [93]. However, most of novel bnAb epitopes (approximately 90%) are non-continuous and constituted regions brought together in 3-dimentional configurations [120]. Although the presentation of discontinuous epitopes onto a protein scaffold could be predicted by computational modeling [121], these bnAb epitopes might be challenged to be embedded in non-enveloped HPV16 L1 protein scaffold. By contrast, a minority of HIV-1 B-cell immunogens, such as MPER (2F5) of HIV-1 gp41 or V3 loop (P18IIB) of gp120, contains linear neutralizing epitopes and might be suitable for the HPV:HIV protein backbone. Therefore, we selected the linear 2F5 neutralizing epitope that is included in an extended T20 peptide of HIV-1 MPER in term of favorable structure for α-helix formation [122]. T20 peptides could be fused and stabilized on L1 capsid scaffolds to elicit neutralizing antibody responses if the native configuration of 2F5 epitope could be presented. In this study, we found that 2F5 nAbs were bound to chimeric HPV:HIV (L1:T20) VLPs in vitro. Moreover, the L1:T20 VLPs can also induce T20-specific binding antibodies in BALB/c mice. There was growing evidence that HIV-1 fusion inhibitory (T20) peptide-induced antibodies have similar properties as anti-HIV-1 fusion peptide Enfuvirtide to bind the hydrophobic trans-membrane T20 residue located in MPER of gp41 during HIV-1 fusion and contribute to viral control [109,123,124]. According to our previous reviews, the rational design of chimeric VLP-based vaccines to induce HPV and HIV-specific neutralizing antibody and CTL responses would always need to be considered in terms of immunogen selection, antigen delivery vectors and prime-boost regimes. In conclusion, this study has shown an alternative mammalian cell-based expression platform and a scalable chromatographic purification method to engineer chimeric HPV:HIV VLPs. We consider that our new purification methods will aid in recovering antigenic HPV:HIV VLPs from mammalian cells towards the goal of reducing time, cost, and labor while increasing the capacity for industrial production., Additionally, we demonstrated HPV16 L1 VLPs can work as a delivery platform to carry HIV-1 peptide antigen, because the insertion of P18I10 or T20 peptides into the DE loop of HPV16 L1 capsid proteins did not affect in vitro stability, self-assembly and morphology of chimeric HPV:HIV VLPs and did not interfere HPV16 L1-specific antibody induction in vivo. On the other hand, chimeric HPV:HIV VLPs could elicit HPV16 and HIV-specific B and T-cell immune responses against both viruses. This report explored a possibility of developing HIV-1 vaccines based on recombinant BCG expressing HIV-1 immunogens and HPV:HIV VLPs, which can be used for childhood HIV-1 immunization. Since the development of an effective chimeric vaccine against HPV16 and HIV-1 is still a challenge, this work contributes a step towards the development of the novel chimeric HPV:HIV VLP-based vaccine platform for controlling HPV16 and HIV-1 infection, which is urgently needed in developing and industrialized countries.

## Data Availability

All data are contained within the article.
